# Red Ginseng Extract Attenuates Kainate-Induced Excitotoxicity by Antioxidative Effects

**DOI:** 10.1155/2012/479016

**Published:** 2012-10-23

**Authors:** Jin-Yi Han, Sun-Young Ahn, Eun-Hye Oh, Sang-Yoon Nam, Jin Tae Hong, Ki-Wan Oh, Mi Kyeong Lee

**Affiliations:** ^1^Research Institute of Veterinary Medicine, Chungbuk National University, Cheongju 361-763, Republic of Korea; ^2^College of Pharmacy, Chungbuk National University, Cheongju 361-763, Republic of Korea; ^3^College of Medicine, Chungbuk National University, Cheongju 361-763, Republic of Korea; ^4^College of Veterinary Medicine, Chungbuk National University, Cheongju 361-763, Republic of Korea

## Abstract

This study investigated the neuroprotective activity of red ginseng extract (RGE, *Panax ginseng*, C. A. Meyer) against kainic acid- (KA-) induced excitotoxicity *in vitro* and *in vivo*. In hippocampal cells, RGE inhibited KA-induced excitotoxicity in a dose-dependent manner as measured by the MTT assay. To study the possible mechanisms of the RGE-mediated neuroprotective effect against KA-induced cytotoxicity, we examined the levels of intracellular reactive oxygen species (ROS) and [Ca^2+^]_*i*_ in cultured hippocampal neurons and found that RGE treatment dose-dependently inhibited intracellular ROS and [Ca^2+^]_*i*_ elevation. Oral administration of RGE (30 and 200 mg/kg) in mice decreased the malondialdehyde (MDA) level induced by KA injection (30 mg/kg, i.p.). In addition, similar results were obtained after pretreatment with the radical scavengers Trolox and *N*, *N*′-dimethylthiourea (DMTU). Finally, after confirming the protective effect of RGE on hippocampal brain-derived neurotropic factor (BDNF) protein levels, we found that RGE is active compounds mixture in KA-induced hippocampal mossy-fiber function improvement. Furthermore, RGE eliminated 1,1-diphenyl-2-picrylhydrazyl (DPPH) radicals, and the IC_50_ was approximately 10 mg/ml. The reductive activity of RGE, as measured by reaction with hydroxyl radical (^•^OH), was similar to trolox. The second-order rate constant of RGE for ^•^OH was 3.5–4.5 × 10^9^ M^−1^·S^−1^. Therefore, these results indicate that RGE possesses radical reduction activity and alleviates KA-induced excitotoxicity by quenching ROS in hippocampal neurons.

## 1. Introduction

*Panax ginseng* C. A. Meyer (Araliaceae) is one of the most widely used medicinal plants, particularly in traditional oriental medicine, for the treatment of various diseases. It has a wide range of pharmacological and physiological actions [1, 2]. Red ginseng extract (RGE) derives from a ginseng plant that has been cultivated for 4–6 years or more and goes through an extensive cleaning, steaming, and drying process [3]. Heat treatment of ginseng at 98–100°C for 2-3 h under high pressure increases the production of nonpolar or lesspolar saponins such as Rg_3_,  Rg_5_,  Rg_6_, Rh_2_, and RK_1_. These improved biologically active ginseng products result from changes in the chemical constituents that occur during steaming treatment [4]. In addition, the content of maltol (3-hydroxy-2-methyl-4-pyrone) is increased by heat processing of ginseng. The antioxidant activity of phenolic compounds is correlated with their chemical structures. The structure-activity relationships of some phenolic compounds (e.g., flavonoids, phenolic acids, and tannins) have previously been studied [5–7]. In general, the free radical scavenging and antioxidant activity of phenolics (e.g., flavonoids and phenolic acids) mainly depends on the number and position of hydrogen-donating hydroxyl groups on the aromatic ring of the phenolic molecules and is affected by factors such as the glycosylation of aglycones and other H-donating groups (–NH, –SH). There have been many reports on antioxidant components that generally focus on phenolic acids [5, 8, 9]. RGE has both stimulatory and inhibitory effects on the central nervous system (CNS) and may modulate neurotransmission [10]. Some studies have reported that RGE has antioxidant, memory enhancing, antihypertensive, and antistress effects [11, 12]. Red ginseng protected smokers from oxidative damage and reduced cancer risk associated with smoking [13]. RGE has been reported to scavenge hydroxyl radicals (^•^OH), DPPH, and superoxide radicals [14]. 

Excitatory amino acids (EEAs) such as glutamate are well known as the primary neurotransmitters that mediate synaptic excitation in the vertebrate CNS [15]. Glutamate has dual actions on CNS neurons, acting as an excitatory neurotransmitter at physiologic concentrations and as a neurotoxic substance when present in excess. Glutamate has also been implicated in the initiation of nerve cell death under conditions of stroke, epilepsy, and other forms of central nervous system insult. Glutamate kills neuronal cells through either a receptor-mediated pathway or the inhibition of cysteine uptake and the oxidative pathway [16]. KA is a glutamate analogue with excitotoxic properties [17, 18]. It is well known that KA induces elevations of intracellular Ca^2+^ and extracellular glutamate levels via coactivation of N-methyl-D-aspartate (NMDA) receptors. Glutamate-evoked Na^+^ influx has also been proposed to contribute to the acute form of neurotoxicity [19, 20]. Hippocampal mossy fiber (MF) sprouting is a potential therapeutic target for epilepsy [21, 22]. The induction of BDNF protein is most evident in the MF pathway [21].

Reactive oxygen species (ROS) are by-products generated by cellular oxidative metabolism. Oxidative stress is a causal, or at least an ancillary, factor in the neuropathology of several adult neurodegenerative disorders [23]. ROS are known to play a role in KA-induced neuronal damage. Accumulating evidence indicates that hippocampal oxidative insults might be involved in KA-induced neurotoxicity *in vivo* [24, 25] and *in vitro*. Direct evidence of free radical generation during KA stimulation of cultured retinal neurons was provided by electron spin resonance (ESR) spectroscopy [26, 27]. ESR is a sophisticated spectroscopic technique that detects free radicals or inorganic complexes in chemical and biological systems [28]. ESR spectroscopy of spin-trapped radicals has become the method of choice for the detection and identification of free radicals formed in biological systems [29, 30]. The spin-trapping technique utilizing nitrones has been applied to the detection of free radicals for over thirty years. Nitrone spin traps are used in ESR studies because they specifically react with free radicals to form a radical adduct with a longer lifetime than the initial free radical. For biological applications, nitrone spin traps such as 5, 5-dimethyl-l-pyrroline-N oxide (DMPO) have been used most frequently [29]. 

Despite the widespread use of RGE, knowledge of its mechanism of action or protective effects on glutamate-mediated toxicity is limited. In this study, to elucidate these issues, we investigated the protective effect and possible mechanism of RGE on kainate-induced excitotoxicity in hippocampal neurons.

## 2. Materials and Methods

### 2.1. Chemicals and Reagents

 RGE was kindly provided by the Korea Ginseng Cooperation (Daejeon, Republic of Korea). RGE yielded 4.37% saponins: the main components of ginsenosides were Rb_1_ (12.6%), Rb_2_ (6.2%), Rc (6.9%), Rd (3.4%), Re (6.4%), Rf (2.1%), Rg_1_ (15.8%), and Rg_3_ (1,4%). Those constituents are well standardized and qualified by the Korea Ginseng Cooperation. KA ((2S,3S,4S)-carboxy-4-(1-methylethenyl)-3-pyrrolidineacetic acid) was purchased from Tocris (Ellisville, MO, USA). BDNF was purchased from Abcam Inc. (Cambridge, MA, USA). The OXYTEK thiobarbituric acid reacting substances (TBARS) assay kit was purchased from Alexis (Farmindale, NY, USA). 6-Carboxy-2′,7′-dichlorofluorescin diacetate (DCFH-DA) and fura-4/AM were purchased from Molecular Probes Inc. (Eugene, OR, USA). DMPO was purchased from Enzo (Plymouth Meeting, PA, USA). Ferrous sulfate (Fe_2_SO_4_·7H_2_O), hydrogen peroxide (H_2_O_2_, 30%), diethylenetriamene pentaacetate (DTPA), DPPH, 6-hydroxy-2,5,7,7-tetramethyl-chromane-2-carboxylic acid (Trolox), DMTU, 3-(4,5-dimethylthiazol-2-yl)-2,5-diphenyl tetrazolium bromide (MTT), and all other chemicals were of high quality and were obtained from Sigma (St. Louis, MO, USA).

### 2.2. Animals

Male ICR mice (Samtako, Osan, Korea) weighing 30–35 g were used for *in vivo* experiments (*n* = 7-8). Animals were housed in acrylic cages (45 cm × 60 cm × 25 cm) with water and food available *ad libitum* under an artificial 12 h light/dark cycle (light on at 7 : 00) and constant temperature (22 ± 2°C). Mice were housed in the departmental room for 1 week before testing to ensure adaptation to the new environment. All experiments involving animals were performed in accordance with the National Institutes of Health Guide for the Care and Use of Laboratory Animals (NIH publication no. 85-23, revised 1985). The Institutional Animal Care and Use Committee of Chungbuk National University approved the protocol.

### 2.3. Primary Hippocampal Neuronal Cell Culture and KA Exposure

 Primary cultures of rat hippocampal neurons were prepared from the hippocampi of E18-19 Sprague-Dawley (SD) rat embryos and cultured according to a previously described method [31]. The hippocampi were dissected and incubated with 0.25% papain in Ca^2+^ and Mg^2+^-free Hank's balanced salt solution at 37°C for 20 min. Cells were then mechanically dissociated with fire-polished Pasteur pipettes by trituration and plated on poly-L-lysine coated coverslips in 35 mm culture dishes. Cells were maintained in Neurobasal/B27 medium containing 0.5 mM L-glutamine, 25 *μ*M glutamate, 25 *μ*M 2-mercaptoethanol, 100 unit/mL penicillin, and 100 *μ*g/mL streptomycin in a humidified atmosphere of 95% air and 5% CO_2_ at 37°C, with half of the medium changed every 2 days. Hippocampal neurons were cultured for 12–14 days before KA (100 *μ*M) exposure. RGE (0.01–1.0 mg/mL) was added 0.5–1 h before KA treatment.

### 2.4. Cell Viability Assay

Cell viability assays were performed as described [32]. After exposure for the indicated times, neurons were assayed for viability using MTT (Sigma, St. Louis, MO, USA), which was added at a final concentration of 5 mg/mL for 4 h. MTT was removed, and neurons were lysed in 200 *μ*L of dimethyl sulfoxide (DMSO). The absorbance was measured at 570 nm on a SpectraMax M2 multimode microplate reader (Sunnyvale, CA, USA). The data are expressed as the percentage of unexposed neurons that remained in the presence of KA.

### 2.5. Intracellular ROS Measurement

Production of ROS in neurons was determined using DCFH-DA (Molecular Probes, Eugene, OR, USA) as previously described [33]. Cultures were incubated with 10 *μ*M DCFH-DA at 37°C for 30 min. After DCFH-DA was removed, the cells were recorded. DCFH-DA-loaded cells were placed in a SpectraMax M2 multiwell fluorescence microplate reader (Sunnyvale, CA, USA) with excitation at 515 nm and emission 552 nm. The protein concentration was determined by the Bradford assay. 

### 2.6. Intracellular Calcium Measurement

The acetoxymethyl ester form of fura-4 (Molecular probes, Eugene, OR, USA) was used as the fluorescent Ca^2+^ indicator. Hippocampal cells were incubated for 60 min at room temperature with 5 *μ*M fura-4/AM and 0.001% Pluronic F-127 in a HEPES-buffered solution composed of (in mM) 150 NaCl, 5 KCl, 1 MgCl_2_, 2 CaCl_2_, 10 HEPES, and 10 glucose. The pH was adjusted to 7.4 with NaOH. The cells were then washed with HEPES-buffered solution and placed on a SpectraMax M2 multiwell fluorescence microplate reader (Sunnyvale, CA, USA). Emitted fluorescence was calculated using a fluorescence analyzer and converted to intracellular free Ca^2+^ concentration [Ca^2+^]_*i*_. 

### 2.7. Lipid Peroxidation Assay

Lipid peroxide formation was analyzed by measuring the TBARS in homogenates, as described by Suematsu et al. [34]. The OXYTEK TBARS assay Kit was used for these measurements. Lipid peroxidation was determined using their protocol by measuring the absorbance at 532 nm and was expressed as nmol of malondialdehyde (MDA)/mg of protein. The protein concentrations of hippocampi were determined using the Bradford assay.

### 2.8. Western Blotting Assay

Cells were harvested, washed twice with ice-cold PBS, and lysed in a lysis buffer for 30 min on ice, with vortexing every 5 min. Lysates were then centrifuged at 14,000 rpm for 5 min to remove insoluble material. Protein concentrations were determined by the Bradford method (Bio-Rad) using BSA as a standard. For BDNF, protein was separated on 16% SDS-PAGE gels. The gels were subsequently transferred onto PVDF membranes (Amersham Hybond TM-P, GE Healthcare, Buckinghamshire, UK) by electroblotting for 2 h at 60–75 V. Membranes were then blocked with 5% nonfat milk solution in Tris-NaCl buffer (TNT) containing 0.5% Tween-20 and incubated with primary antibodies as indicated. Monoclonal donkey anti-rabbit IgG horseradish peroxidase-conjugated secondary antibodies were used at 1 : 3,000. Proteins were detected by enhanced chemiluminescence using a commercial kit (Amersham).

### 2.9. 1,1-Dipheny-2-picrylhydrazyl (DPPH) Assay

 The scavenging of the stable free radical DPPH by RGE was assayed spectrophotometrically [35]. DPPH in ethanol (0.1 mM) (control) was mixed thoroughly with various concentrations of RGE (0–10 mg/mL), and the absorbance was read at 517 nm. The degree of DPPH radical scavenging activity of RGE was calculated as a percentage of inhibition (% inhibition), where

(1) $$\begin{array}{c} \%\;\text{inhibition}=\;\left[\frac{\left(A_{\text{control}}-A{}_{\text{sample}}\right)}{A_{\text{control}}}\right]\times 100. \end{array}$$

### 2.10. ^•^OH Scavenging Activity by ESR

^•^OH was generated by the Fenton Reaction System, and the generated ^•^OH rapidly reacted with the nitrone spin trap DMPO [29]. The resultant DMPO/^•^OH adduct was detected with an ESR spectrometer. RGE (0.2 mL) at various concentrations was mixed with DMPO (0.2 M, 0.2 mL), Fe_2_SO_4_ (2.0 mM, 0.2 mL), and H_2_O_2_ (2.0 mM, 0.2 mL) in a phosphate buffer solution (100 mM, pH 7.2), and the mixture was transferred to a quartz flat cell for ESR measurements. The mixture was performed in an ESR cavity at room temperature (24-25°C). After reaction, the ESR spectrum was recorded at room temperature using an ESR (JEOL JESTE-300) spectrometer (JEOL, Inc., Tokyo, Japan) equipped with a TE_102_ cavity. Experimental conditions were as follows: magnetic field, 339.3 ± 10 mT; power, 2.2 mW; modulation frequency, 9.44 GHz; amplitude, 10 × 10; sweep time, 0.5 min. The results were indicated as the time required to produce a 50% inhibition or decrease in signal peak height (IH_50_) by ESR.

### 2.11. Statistical Analysis

Data were presented as means ± SEM. For statistical comparisons, the results were analyzed using one-way analysis of variance (ANOVA). A *P* value < 0.05 was considered statistically significant. In the case of significant variation, the individual values were compared with the Holm-Sidak test.

## 3. Results

### 3.1. Protection from Kainate Toxicity by RGE

**Figure 1:**
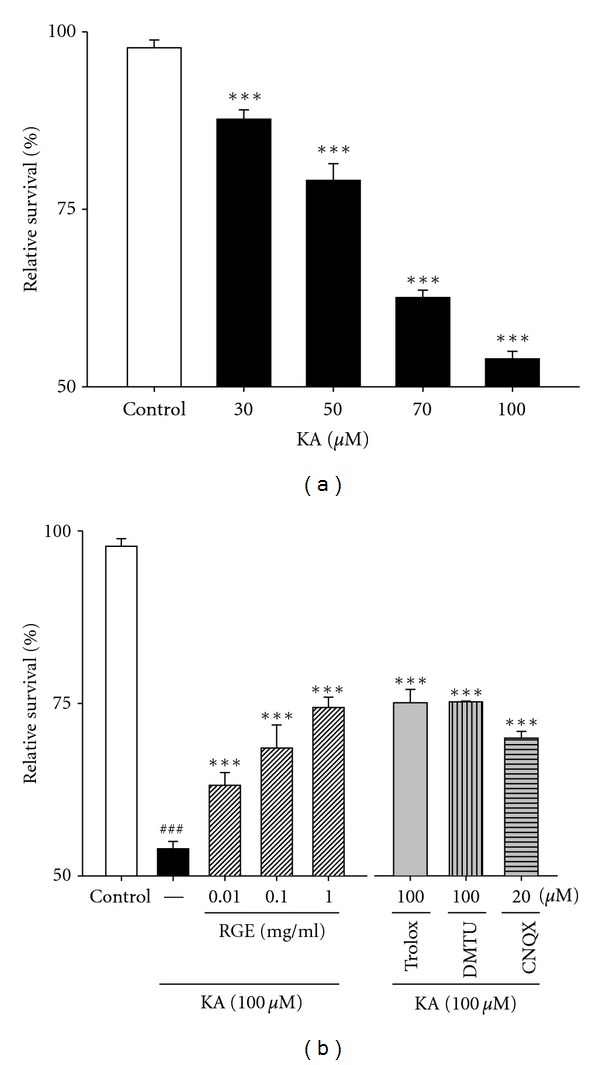
RGE prevents KA-induced neuronal loss in primary cultured hippocampal cells. (a) Concentration data for KA-induced toxicity in primary cultured hippocampal neurons. Examination of the dose effect of KA on neuronal viability by the MTT assay. Neurons were exposed to KA at concentrations of 0, 30, 50, 70, and 100 *μ*M for 48 h. (b) Protection of hippocampal neurons against KA-induced cell loss by RGE. Neurons were exposed to 100 *μ*M KA at 1 h after 0.01–1.0 mg/mL of RGE, Trolox (100 *μ*M), or DMTU (100 *μ*M) treatment. Cell viability at 48 h after KA exposure was measured by the MTT assay. All data are presented as means ± SE. ^###^ *P* < 0.001 versus the control group. ***P < 0.001 versus the KA group.

**Figure 2:**
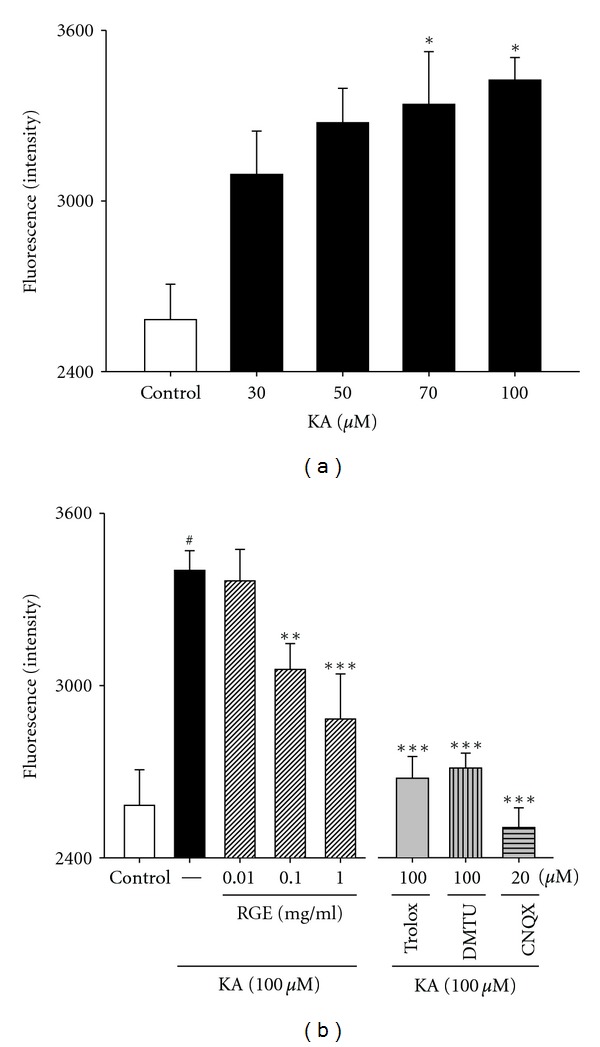
Effects of RGE and scavengers against oxidative stress in primary cultured hippocampal cells. (a) Concentration data for KA-induced ROS levels in primary cultured hippocampal neurons. Examination of the dose effect of KA on neuronal ROS level by the DCFH-DA assay. Neurons were exposed to KA at concentrations of 0, 30, 50, 70, and 100 *μ*M for 48 h. (b) Protection of hippocampal neurons against KA-induced ROS levels by RGE. Neurons were exposed to 100 *μ*M KA at 1 h after 0.01–1 mg/mL of RGE, trolox (100 *μ*M), or DMTU (100 *μ*M) treatment. All data are presented as means ± SE. ^###^ *P* < 0.001 versus the control group. *P < 0.05, **P < 0.01, ***P < 0.001 versus the KA group.

To evaluate the protective effect of RGE against KA-induced cytotoxicity, we examined cell death in primary hippocampal neurons by the MTT assay. Neurons were exposed to KA at concentrations of 0, 30, 50, 70, and 100 *μ*M for 48 h (Figure 1(a)).  Figure 2 shows that cell death is rapid, with growth inhibited to 55.1 ± 1.0% of control levels after 48 h of KA exposure. When cells were exposed to 100 *μ*M KA, cell death was significant after 48 h (Figure 1). Exposure of hippocampal neurons to 100 *μ*M of KA for 48 h elicited a significant decrease in cell survival, whereas KA-induced neuronal loss was inhibited by 63.1 ± 1.8% and 74.4 ± 1.4% by adding 0.01 and 1.0 mg/mL of RGE, respectively (Figure 1). Additionally, 76.8 ± 1.9% and 76.9 ± 0.1% inhibition of cell death were achieved by 100 *μ*M trolox and DMTU, respectively. When we explored the role of non-N-methyl-D-aspartate (NMDA) receptors in excitotoxicity, KA-induced cell death was blocked by 20 *μ*M 6-cyano-7-nitroquinoxaline-2,3-dione (CNQX), a non-NMDA receptor antagonist. These results indicate that RGE can protect neurons against KA-induced cytotoxicity.

### 3.2. Effects of RGE against Oxidative Stress

Because KA induces oxidative damage in cultured murine neurons, we examined whether RGE affected 100 *μ*M KA-induced ROS levels in primary hippocampal neuronal cells by the DCFH-DA assay. Low levels of ROS were found in control (2, 582 ± 124 fluorescence intensity), and these values were considered physiological. In contrast, a significant increase in ROS concentration was seen, with an intensity value of 3, 424 ± 79, after treatment with 100 *μ*M KA for 48 h. As shown in Figure 2, ROS levels displayed intensity values of 3, 056 ± 89 and 2, 883 ± 157 at doses of 0.1 and 1.0 mg/mL of RGE, respectively. Trolox and DMTU also exhibited an inhibition in ROS production at the highest KA dose of 100 *μ*M (Figure 2). Taken together, these results indicate that 100 *μ*M KA treatment elevates ROS production and that pretreatment with 0.1 to 1.0 mg/mL of RGE significantly decreases ROS production (Figure 2). These results indicate that RGE can protect neurons against KA-induced excitotoxicity through its antioxidant effects. 

### 3.3. Inhibition of Ca^2+^ Influx

**Figure 3:**
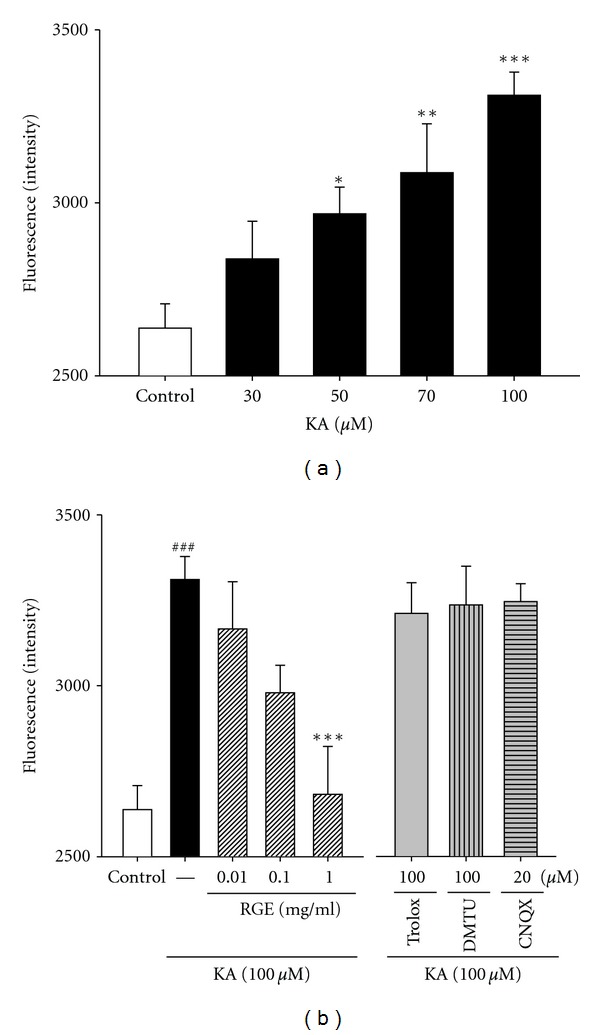
Effect of RGE and scavengers on [Ca^2+^]_*i*_ influx in primary cultured hippocampal cells. (a) Concentration data for KA-induced [Ca^2+^]_*i*_ in primary cultured hippocampal neurons. Examination of the dose effect of KA on neuronal [Ca^2+^]_*i*_ by the Ca^2+^ indicator with fura-4. Neurons were exposed to KA at concentrations of 0, 30, 50, 70, and 100 *μ*M for 48 h. (b) Protection of hippocampal neurons against KA-induced [Ca^2+^]_*i*_ by RGE. Neurons were exposed to 100 *μ*M KA at 1 h after 0.01–1.0 mg/mL of RGE, trolox (100 *μ*M), or DMTU (100 *μ*M) treatment. All data are presented as means ± SE. ^###^ *P* < 0.001 versus the control group. *P < 0.05, and **P < 0.01, ***P < 0.001 versus the KA group.

 In oxidative glutamate toxicity, a 100-fold increase in intracellular ROS results in the elevation of cytosolic Ca^2+^, which precedes cell death. To investigate the mechanism of protection of RGE against KA-induced neurotoxicity, we examined whether RGE could inhibit KA-induced intracellular [Ca^2+^]_*i*_ elevation in cultured hippocampal neurons. We measured [Ca^2+^]_*i*_ levels using the Ca^2+^ indicator, fura-4. As shown in Figure 3, 100 *μ*M KA treatment led to a significant elevation of [Ca^2+^]_*i*_ (data not shown). The % inhibition of [Ca^2+^]_*i*_ elevation by RGE in hippocampal neurons was 4.4 ± 0.04%, 10.1 ± 0.02%, and 18.9 ± 0.04%, at doses of 0.01, 0.1, and 1.0 mg/mL, respectively, where [Ca^2+^]_*i*_ levels in normal controls were considered 100% (Figure 3). Treatment with RGE at doses of 0.01, 0.1, and 1.0 mg/mL inhibited KA-induced [Ca^2+^]_*i*_ elevation in a dose-dependent manner. In contrast, treatment with trolox, DMTU, or CNQX made slight effect. These results indicate that 100 *μ*M KA treatment of cultured hippocampal neurons elevates [Ca^2+^]_*i*_ and that this effect is attenuated by treatment with RGE at concentrations of 0.01, 0.1, and 1.0 mg/mL. 

### 3.4. Inhibition of MDA Levels by RGE

**Figure 4:**
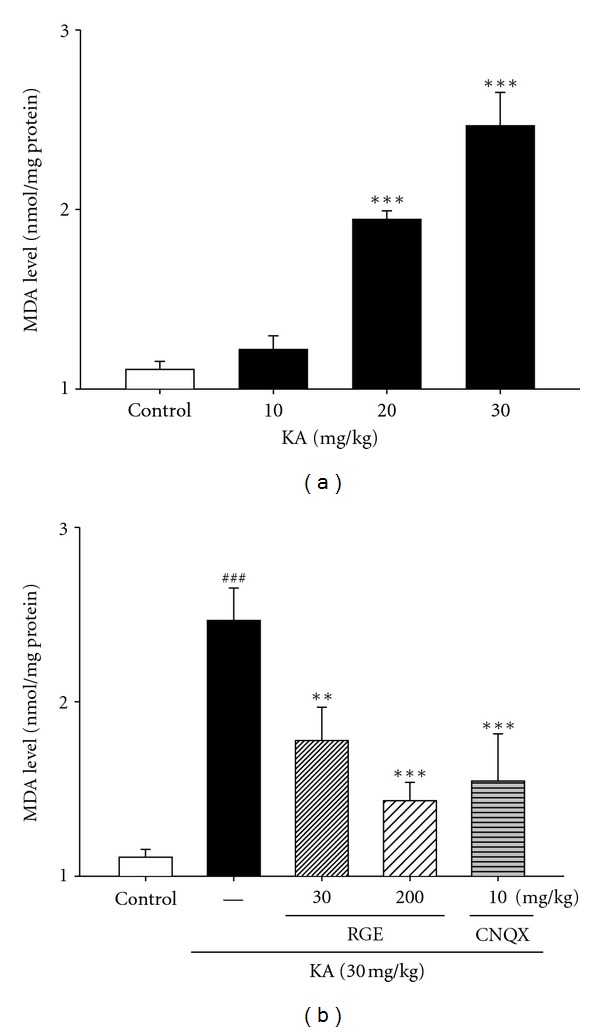
Effect of RGE and scavengers on MDA levels in KA-treated hippocampal tissue homogenates. Male mice were grouped (*n* = 5 or 6/group) and pretreated (i.p. injection) with RGE (30–200 mg/kg) and scavengers such as trolox (50 mg/kg, i.p.) and DMTU (50 mg/kg, i.p.), or NaCl (0.9%). Thirty minutes after the final RGE or saline pretreatment, seizures in the KA, scavengers + KA, and RGE + KA groups were induced by KA injection (30 mg/kg, i.p.); the mice in the saline group received an equal volume of 0.9% NaCl. All data are presented as means ± SE. ^###^ *P* < 0.001 versus the control group. **P < 0.01, ***P < 0.001 versus the KA group.

 Free radicals may be one of the major causes of excitotoxicity lesions. Therefore, we estimated free radical generation using a TBARS assay. TBARS levels, an indicator of lipid peroxidation, were significantly increased in the hippocampi of mice treated with KA dose-dependently when compared to the control group (vehicle) that had not received the stressor agent. However, pretreatment with 30 and 200 mg/kg doses of RGE for 10 days significantly prevented KA-induced increase in TBARS levels. Complete inhibition of lipid peroxidation was observed at 200 mg/mL of RGE (Figure 4). Animals treated only with RGE at both doses presented no alteration in TBARS levels (data not shown).

### 3.5. DPPH Radical Reduction Activity of RGE

**Figure 5:**
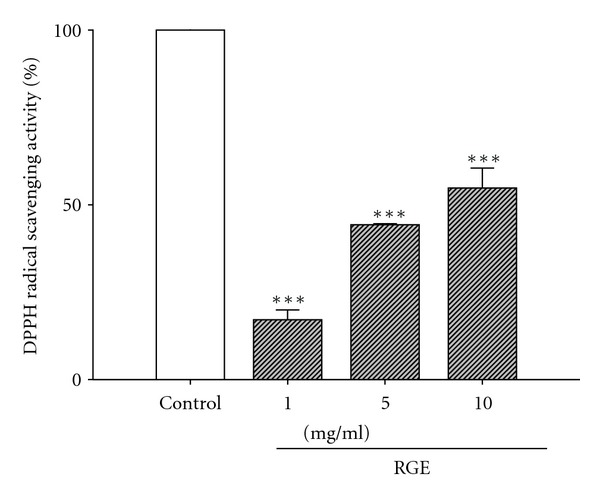
DPPH radical-scavenging activity of RGE. 0.01–1.0 mg/mL of RGE was used. Trolox, as a positive control, scavenged 100% of the DPPH radical at 0.25 mg/mL. All data are presented as means ± SE. ***P < 0.001 versus the control group.

The activity of ginseng is generally explained by its antioxidative efficacy. To identify the redox potential of RGE, the reduction of the DPPH radical was analyzed in mixed solutions (RGE+DPPH).  Figure 5 shows that RGE scavenged the DPPH radical in a dose-dependent manner, and the IC_50_ of RGE was approximately 10 mg/mL (Figure 5). Trolox used as a positive control scavenged 100% of the DPPH radical at 0.25 mg/mL.

### 3.6. ^•^OH Reduction Activity of RGE

**Figure 6:**
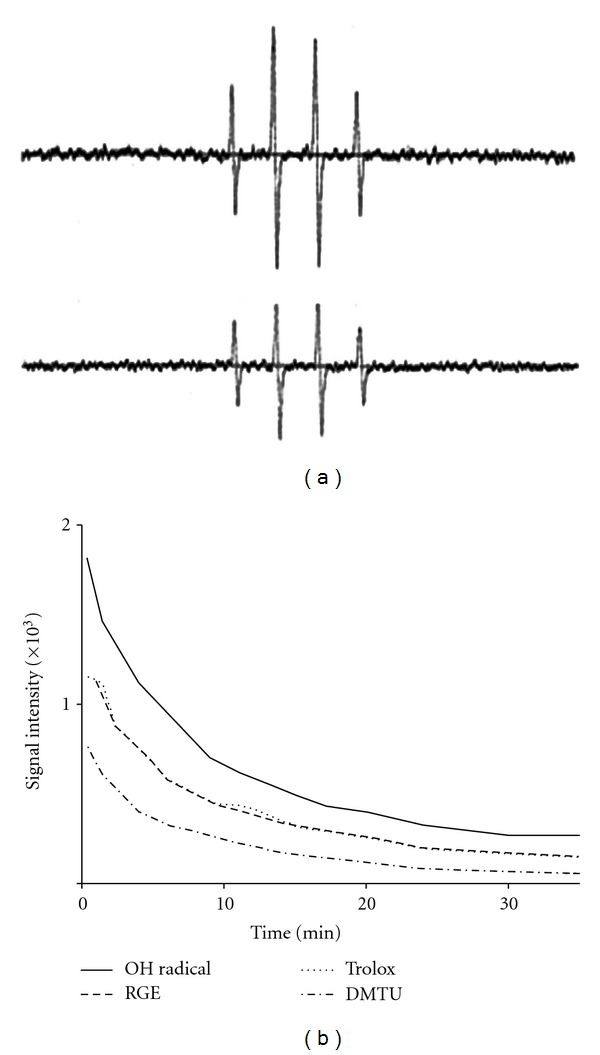
Reduction activity of RGE on ^•^OH. (a) ESR spectra of the DMPO/^•^OH adduct were generated in a Fenton Reaction System. The solutions with a final volume of 0.1 mL contained 2.0 mM ferrous sulfate, 2.0 mM H_2_O_2_, and 100 mM phosphate buffer (pH 7.2). Reactions were started by the addition of ferrous ammonium sulfate (2.0 mM final concentration), and the steady-state ESR spectra were recorded at 15 min after the Fenton Reaction. Upper line, DMPO/^•^OH adduct; lower line, RGE (1.0 mg/mL). (b) Time course of ^•^OH degradation induced by the Fenton Reaction System. DMPO/^•^OH adduct, RGE (1.0 mg/mL), trolox (1.0 mM), and DMTU (1 mM).

^•^OH generated by the Fenton Reaction System was trapped by DMPO, which could be detected by an ESR spectrometer. The typical 1 : 2 : 2 : 1 ESR signal of the DMPO/^•^OH adduct (*A*_N_ = *A*_H_ = 14.4 G) was observed as shown in Figure 6(a). Each spectrum was obtained 15 min after the start of the Fenton Reaction. RGE inhibits the Fenton Reaction by reaction with ^•^OH. In addition, as shown in Figure 6(b), the signal of the DMPO/^•^OH adduct gradually decreased over time. The decay rate showed approximately a pseudo-first-order kinetics over the period of measurement (10 min), and the half-life of the DMPO/^•^OH signal was estimated to be 8.15 min. The order of reduction activities in the Fenton Reaction System was DMTU > RGE > trolox. The RGE reduction activity was similar to trolox. The activities of DMTU, RGE, and trolox were 5.76, 7.5–8, and 8.17 min, respectively. The second-order rate constants were estimated to be 3.4 × 10^9^ M^−1^·S^−1^ for trolox and 4.7 × 10^9^ M^−1^·S^−1^ for DMTU. From these results, it is possible to estimate the apparent second-order rate constant of RGE for ^•^OH. Therefore, we demonstrated that RGE reacts with ^•^OH and has reduction activity and that the second-order rate constant of RGE for ^•^OH is approximately 3.5–4.5 × 10^9^ M^−1^·S^−1^. 

### 3.7. Alterations in BDNF Protein Levels after KA Injury

**Figure 7:**
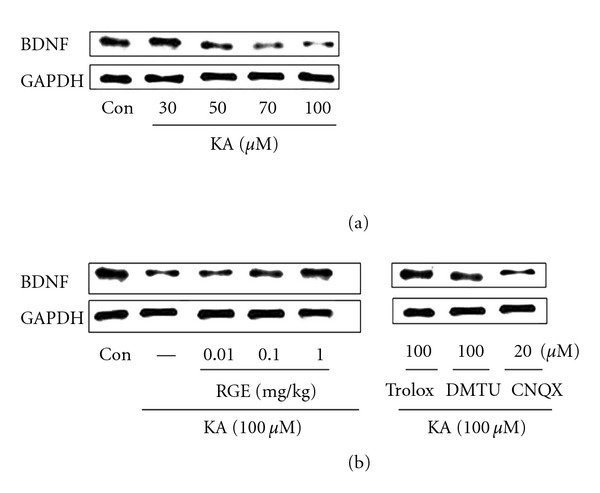
Effect of RGE and scavengers on BDNF levels in KA-treated hippocampal neurons. Immunoblots of lysed embryonic rat hippocampi 2 days following administration of RGE or KA are shown. Neurons were exposed to KA at concentrations of 0, 30, 50, 70, and 100 *μ*M for 48 h. Neurons were exposed to 100 *μ*M KA at 1 h after 0.01–1.0 mg/mL of RGE, trolox (100 *μ*M), and DMTU (100 *μ*M) treatment. GAPDH levels were measured to confirm equal protein loading.

In search of endogenous substances having protective action against EAA, we investigated alterations in hippocampal BDNF expression induced by KA (Figure 7). BDNF protein was decreased after KA exposure. Downregulation of hippocampal BDNF protein induced by KA was increased above control levels by RGE treatment. Decrease in BDNF expression in response to KA was antagonized by CNQX. Moreover, slight changes in BDNF expression were noted in trolox and DMTU treatment. Collectively, these data suggest that RGE is an active compound in KA-induced hippocampal mossy-fiber function improvement.

## 4. Discussion

Our results clearly demonstrate that RGE, one of the most widely used medicinal plants, has significant antioxidant/neuroprotective effects against kainic acid- (KA-) induced excitotoxicity *in vitro* and *in vivo. *We found that RGE reduced* in vitro* toxicity induced by KA, a potent neurotoxin. KA inhibited cell death and the generation of [Ca^2+^]_*i*_ and ROS in hippocampal neurons and increased MDA and BDNF levels in hippocampal tissue. In addition, RGE possesses ^•^OH reduction activity in the Fenton Reaction System as measured using the ESR spectrometer.

KA is a specific agonist of the KA receptor and a selective ionotropic glutamate agonist. Glutamate toxicity is the major contributor to pathological cell death within the nervous system and appears to be mediated by ROS [36]. Oxidative glutamate toxicity has also been implicated in the initiation of nerve cell death under conditions of stroke, epilepsy, and other forms of central nervous system insult. ^•^OH is a strong oxidant. ESR is the preferred tool for detecting and identifying free radicals and is widely used. The ESR spin-trapping technique is the only viable spectroscopic technique for detecting ^•^OH under physiologically relevant conditions [29, 30, 37]. For ^•^OH signal detection, we used the Fenton Reaction System, as shown in Figure 6. RGE inhibits the Fenton Reaction by reaction with ^•^OH because of its fast initial velocity and high second-order rate constant. DPPH and ESR spin-trapping data are good pieces of evidence that indicate the radical reduction activity of RGE (Figures 5 and 6) [38, 39]. Additionally, we estimated the second-order rate constant of RGE/^•^OH to be 3.5–4.5 × 10^9^ M^−1^·S^ −1^.

The mammalian hippocampus plays a pivotal role in a diverse set of cognitive functions, including memory. Glutamatergic signaling in the hippocampus changes during oxidative stress. Cultured rat hippocampal neurons are a useful model for studying the glutamate system. In the toxicity experiments shown in Figure 1, RGE exerted neuroprotective effects against glutamate-induced neurotoxicity. These effects may be explained by the redox antioxidant system, although we cannot rule out the involvement of other mechanisms. Antioxidants in foods and medicinal plants (or herbs) have attracted interest in recent years. Ginsenosides, the main pharmacologically active constituents of ginseng, consist of four hydrophobic ring steroid-like structures with hydrophilic sugar moieties. Free, monomeric, dimeric, or trimeric sugars are bound to hydroxyl groups (^•^OH) on carbon-3, -6, and -20 of ginsenosides. They also exist as stereoisomers. This epimerization is known to occur by the selective attack of ^•^OH after the elimination of the glycosyl residue at C-20 during the steaming process. In addition, less-polar ginsenosides such as RGE are known to be easily produced by the elimination of H_2_O at C-20 of the RG species under high pressure and temperature conditions as in autoclaving. Phenolic compounds are commonly found in plants and have been reported to have multiple biological effects, including antioxidant activity [8, 40, 41]. Phenolic compounds and maltol have strong free radical scavenging activities [42, 43]. In addition, the chelating activity of the hydroxypyrone structure, like other iron chelators such as desferrioxamine (DFO), is very important. Based on these reasons, it is possible that the activity of the phenol extract of RGE is due to its hydrogen-donating ability or chelating ability.

Intracellular calcium levels are usually very low (~10^−7 ^M). Excessive accumulation of intracellular calcium is the key process leading to neuronal death or injury. NMDA receptors activate channels that allow the influx of extracellular calcium (and sodium) [44]. Overstimulation of this type of glutamate receptor leads to neuronal calcium overload. Both AMPA and KA receptors are linked to Na^+^ permeable channels. Depolarization is initiated primarily by activation of AMPA receptors and subsequently by activation of voltage-dependent sodium channels. This leads to sodium entry and further depolarization. Na^+^ influx can be involved in the toxic process by causing osmotic stress [20] and via depolarization by opening voltage-operated calcium channels. In addition, Ca^2+^ influx can damage neurons by activating various enzymes. If the cell becomes chronically depolarized, the NMDA receptor will relinquish its magnesium block and become available for activation by synaptic glutamate. Its activation is a major source of calcium entry into the cell. Sustained increase in intracellular Ca^2+^ concentration [Ca^2+^]_*i*_ initiates the excitotoxic processes culminating in delayed neuronal death. 

KA is toxic mainly in the hippocampus, which has high-affinity KA binding sites. To search for endogenous substances having a protective effect against EAA, we investigated the alterations in hippocampal BDNF expression. The protein family of neurotrophins consisting of nerve growth factor (NGF), BDNF, NT-3, NT-4/5, NT-6, and NT-7 is known to regulate the survival and differentiation of peripheral nervous system (PNS) and CNS neurons [45]. In the recent years, evidence has accumulated that BDNF plays an additional important role in hippocampal synaptic plasticity by either facilitating transmitter release from presynaptic terminals or enhancing postsynaptic NMDA receptor function. Therefore, it is possible that the attenuation of NMDA neurotoxicity is caused by diffusible factors secreted by the striatal cells, such as neurotrophins like BDNF. The substances protecting neurons from stress caused by ROS such as ^•^OH may have a role in the promotion of hippocampal mossy-fiber functions in the CNS.

## 5. Conclusions

RGE protected hippocampal neurons from toxicity due to KA-induced increases in [Ca^2+^]_*i*_, ROS, and MDA levels. The RGE reaction with DPPH and ^•^OH possesses radical reduction activity, and the second-order rate constant for ^•^OH is 3.5–4.5 × 10^9^ M^−1^·S^−1^. RGE alleviates KA-induced excitotoxicity by quenching ROS in hippocampal neurons.
